# Development of a multiplex fluorescent qPCR assay for the simultaneous detection of bovine viral diarrhea virus and pathogenic *Escherichia coli*

**DOI:** 10.1371/journal.pone.0349315

**Published:** 2026-05-15

**Authors:** Wenxin Zhu, Huanxing Zhu

**Affiliations:** 1 Pharmaceutical Chemistry Teaching & Research Office, Binzhou Medical University, Yantai, China; 2 Zhucheng Animal Husbandry Development Center, Weifang, China; Cairo University Faculty of Veterinary Medicine, EGYPT

## Abstract

Neonatal calf diarrhea, primarily caused by bovine viral diarrhea virus (BVDV) and enterotoxigenic *Escherichia coli* K99, poses a significant threat to the cattle industry. This study aimed to develop a multiplex qPCR assay for the simultaneous detection of both pathogens. We established specific primers and probes and generated optimized qPCR standard curves, achieving high analytical sensitivity, with limits of detection of 10² copies/μL for the BVDV target fragment and 10¹ copies/μL for the *E. coli* K99 plasmid DNA, without cross-reactivity with other bovine pathogens. The assay demonstrated good repeatability and was validated using clinical samples, confirming its effectiveness for accurate diagnostics. This multiplex qPCR assay enhances monitoring of BVDV and *E. coli* infections in livestock, potentially reducing economic losses from outbreaks.

## Introduction

Neonatal calf diarrhea (NCD), a common cause of growth impairment and mortality in calves, causes significant economic losses in the livestock industry [1]. According to the National Animal Health Monitoring System (NAHMS) of the United States dairy industry, calf diarrhea is responsible for 57% of the mortality observed in pre-weaned calves. Furthermore, a calf mortality rate of 20% can reduce net income by 38% [2]. As the scale of livestock farming continues to expand, NCD not only results in stunted growth but also causes dehydration and death, severely affecting the healthy development of the cattle industry. Among the pathogens responsible for calf diarrhea, bovine viral diarrhea virus (BVDV) and *Escherichia coli* are particularly notable due to their high prevalence and severe impact on livestock production.

BVDV is a single-stranded RNA virus of the *Flaviviridae* family and *Pestivirus* genus, with a 12.3-kb genome that contains a single open reading frame (ORF) flanked by 5′ and 3′ untranslated regions (UTRs). The 5′UTR is used for genotyping and subtyping BVDV [3,4]. BVDV is classified into three genotypes—BVDV-1, BVDV-2, and BVDV-3—and infects cattle and other animals, such as goats, sheep, and pigs [5,6]. Symptoms of BVDV infection include diarrhea, respiratory issues, immunosuppression, and reproductive problems, all of which significantly affect cattle health and productivity [7,8]. In utero infection can result in immune tolerance and persistently infected (PI) animals, which are key sources of virus transmission [9].

*E. coli* is a facultative anaerobic bacterium commonly found in the intestines of warm-blooded animals. It is one of the leading causes of calf diarrhea and septicemia, and as such, is closely related to the production of dairy products and beef [10,11]. The virulence determinants and distinct pathotypes of this microorganism define several diarrheagenic *E. coli* (DEC) pathotypes, including enteropathogenic (EPEC), enterohemorrhagic (EHEC/STEC), enteroaggregative (EAEC), enterotoxigenic (ETEC), and enteroinvasive (EIEC) *E. coli* [12,13]. *E. coli* detection is part of the routine epidemiological surveillance for calf diseases and serves as a key diagnostic indicator for calf diarrhea. Furthermore, calves can act as important hosts for human pathogenic *E. coli*, posing a potential risk to human health [14]. K99 is a fimbrial adhesion protein that facilitates the attachment of ETEC to the mucosal surfaces of the intestines of calves, lambs, and piglets, enabling the bacterium to colonize the intestinal epithelium and establish infection [15].

BVDV and *E. coli* are key pathogens in calf diarrhea, and coinfection with these pathogens causes immunosuppression and gastrointestinal damage, leading to severe health issues and economic losses. Therefore, early diagnosis and monitoring of BVDV and *E. coli* coinfections are of great importance in reducing the economic losses in the livestock industry. Numerous studies have reported diagnostic methods for BVDV and *E. coli*, including pathogen-based, serological, and molecular techniques [16]. Molecular methods such as quantitative reverse transcription PCR (qRT-PCR), droplet digital PCR (ddPCR), loop-mediated isothermal amplification (LAMP), and recombinase polymerase amplification (RPA) are widely used due to their sensitivity and convenience, but most of these assays only detect a single pathogen. There are limited studies reporting the development of dual-detection methods for both BVDV and *E. coli*. With technological advances, multiplex qPCR has gained popularity for its high sensitivity, specificity, and speed in detecting multiple targets, especially in meat and viral detection [17]. Although diagnostic assays targeting BVDV or *Escherichia coli* individually are already available, neonatal calf diarrhea is frequently associated with mixed infections involving viral and bacterial pathogens. In routine clinical practice, separate testing for individual pathogens increases diagnostic time, cost, and sample consumption, and may delay appropriate intervention. Therefore, a rapid and simultaneous detection method targeting both major pathogens is of significant practical value. Accordingly, we developed a dual-target qPCR assay for the simultaneous detection of both pathogens. This assay provides enhanced sensitivity, reduced testing time, and the ability to detect coinfections in a single assay, which is ideal for large-scale cattle screening and herd health management.

## Materials and methods

### Design and synthesis of specific primers and probes

The primers and probe were designed based on the genomic sequences of BVDV (GenBank No.: ON901784.1) and the *E. coli* K99 gene (GenBank No.: M35282.1). To ensure broad reactivity, fifteen representative sequences for each target (S1 Table) were retrieved from GenBank and aligned using MEGA-X (Molecular Evolutionary Genetics Analysis X) software. Multiple sequence alignment of representative sequences was performed to evaluate the conservation of primer and probe binding regions (S1 Fig). Primers and probes were designed within highly conserved regions identified through multiple-sequence alignment. The primers targeted the 5′ UTR of BVDV and the conserved K99 gene region of *E. coli*, and were designed using Primer Premier 5.0 software, with subsequent verification of specificity using Primer–BLAST. The selected primers and probes were synthesized by Sangon Biotech (Shanghai, China) Co., Ltd. Table 1 shows the details of the primers and probes used in this study.

**Table 1:** Primer and probe sequences for the detection of Bovine Viral Diarrhea Virus (BVDV) and *Escherichia coli* K99 gene. The primers were designed based on the 5′ untranslated region (5′-UTR) of BVDV (GenBank No.: ON901784.1) and the conserved K99 gene region of *E. coli* (GenBank No.: M35282.1). Details on the primers used for BVDV and *E. coli* in the multiplex qPCR assay.

Primer and Probe	Sequence（5’ −3’）	5’ Label	3’ Label
BVD-UTR-F1	CTCAGCGAAGGCCGAAAA	FAM	BHQ1
BVD-UTR-R1	GACTACCCTGTACTCAGGGCTT		
BVD-UTR-T1	AGGCTAGCCATGCCCTTAGTAGGA		
BVD-UTR-F2	GATGGCTGAAGCCCTGAGT	FAM	BHQ1
BVD-UTR-R2	GTCCACGTGGCATCTCGAG		
BVD-UTR-T2	CAGGGTAGTCGTCAGTGGTTCGA		
EC-K99-F1	TCGTTATTTTGCCATTGAAGTTA	HEX	BHQ1
EC-K99-R1	TTTTGCGACTACCAATGCTTC		
EC-K99-T1	CAAGTAGCACTCGTTATTTTGCC		
EC-K99-F2	CCTGAGGTCAATGGTAATCGTA	HEX	BHQ1
EC-K99-R2	CCACTACAGTGCCATGACCA		
EC-K99-T2	TTTCATCTAGATTCTCCATTGTTCGA		
EC-K99-F3	CCAATGCTTCTGCGAATACAGGT	HEX	BHQ1
EC-K99-R3	TGCGAATACAGGTACTATTAACTTCA		
EC-K99-T3	TAATAGCAGCCTGCCCAAGATCTA		

### Construction of the standard reference plasmids

Gene fragments were based on the GenBank accession numbers for the BVDV 5′ UTR fragment and the K99 gene of *Escherichia coli*. Gene fragments covering the fluorescent quantitative amplification region were selected and cloned into the pUC57 plasmid vector to construct the standard plasmids. These plasmids were synthesized by General Biology (Anhui, China) Co., Ltd. and were named pUC57-BDV-UTR and pUC57-EC-K99. The plasmid copy number was calculated using the following formula: Copy number (copies/μL) = [Concentration (ng/μL) × 10 ⁻ ⁹ × 6.022 × 10²³] / [Plasmid length (bp) × 660] where 6.022 × 10²³ is Avogadro’s number, 660 represents the average molecular weight of one base pair, and 10 ⁻ ⁹ is the conversion factor from nanograms to grams. Based on the copy number calculation formula, the plasmid concentration for each plasmid was diluted to 10^8^ copies/μL for subsequent use.

### Screening of the specific primers and probes

Primers and probes were reconstituted to a final concentration of 100 μM in TE buffer. The primer and probe combinations were prepared as detailed in Table 2. The primer-probe combination system was prepared by mixing 70 μL of TE buffer, 12 μL of the appropriate upstream and downstream primers, and 6 μL of the appropriate probe, and water was added to bring the final volume of 100 μL. Each primer-probe combination system was then thoroughly mixed and stored at −80°C in our laboratory. For the qPCR reaction, a 384-well plate was used. Each well was numbered for amplification using the HIScript II One Step qRT-PCR Probe Kit (Cat. Q222-01, Vazyme Biotech, Nanjing, China). Amplification was performed in a real-time PCR system using the following thermal cycling conditions: 55℃ for 15 min, 95°C for 5 min (initial denaturation), followed by 45 cycles at 95°C for 15 s (denaturation) and 60°C for 30 s (annealing/extension), with fluorescence detection in the FAM and HEX channels. The qPCR amplification setup is provided in Table 3. All experiments were conducted in triplicate to optimize the dual TaqMan RT-qPCR system and reaction conditions. The developed multiplex qPCR assay used the pUC57-BDV-UTR and pUC57-EC-K99 plasmids (10^6^ copies/μL) as the positive controls while double-distilled water was used as the negative control. Because BVDV is an RNA virus and *Escherichia coli* is a DNA bacterium, a one-step RT-qPCR system was used. The reverse transcription step was required only for the BVDV RNA template, whereas the DNA target (E. coli K99 gene) was directly amplified during the PCR phase of the reaction. Conventional RT-PCR for BVDV detection was performed using previously reported primers targeting the 5′UTR region [18]. PCR detection of *Escherichia coli* K99 was performed using primers targeting the K99 fimbrial gene (S2 Table) [19].

**Table 2:** Primer and probe combinations for detecting BVDV and *E. coli* K99 gene, prepared at 100 μM in TE buffer. Information on primer and probe combinations.

Primer-Probe Combination	Forward Primer	Reverse Primer	Probe
BVD-UTR-1	BVD-UTR-F1	BVD-UTR-R1	BVD-UTR-T1
BVD-UTR-2	BVD-UTR-F2	BVD-UTR-R2	BVD-UTR-T2
EC-K99-1	EC-K99-F1	EC-K99-R1	EC-K99-T1
EC-K99-2	EC-K99-F2	EC-K99-R2	EC-K99-T2
EC-K99-3	EC-K99-F3	EC-K99-R3	EC-K99-T3

**Table 3:** Components and volumes for the qPCR amplification system. Detailed information on the amplification system.

Component	Volume
Template (Plasmid,10^5^ copies/μl)	2µl
Primer/Probe Mix	1µl
2 × PCR Mix	10µl
RT-PCR Enzyme	1µl
Ultrapure Water	Fill up to 20 μl

### Establishment of a standard curve for the multiplex qPCR assay

A standard curve was generated using six tenfold serial dilutions of recombinant plasmids ranging from 10¹ to 10⁶ copies/μL. Fluorescent quantitative PCR reactions were performed using these standard plasmids as templates, and negative controls were included in each experiment. All samples and controls were tested in triplicate. The linearity of the qPCR data was analyzed using Roche480 software. A standard curve was plotted to assess the correlation between the cycle threshold (Ct) values and plasmid concentrations [20].

### Evaluation of the specificity of the qPCR assay and clinical sample testing

The specificity of the multiplex qPCR assay was assessed using positive clinical samples of BVDV and *E. coli*, which were preserved in our laboratory, along with nucleic acids from nine other common bovine-related viruses and bacteria including Bovine rotavirus(BRV), Bovine coronavirus(BCoV), Bovine Herpesvirus(BHV), Bovine Respiratory Syncytial Virus(BRSV), Parainfluenza Virus Type 3(PI-3), Bovine Adenovirus Type 3(BAV-3), *Pasteurella multocida*, *Streptococcus bovis* and *Mannheimia varigena*. These non-target pathogens were selected based on their clinical relevance in bovine diseases and their potential to co-occur with the target pathogens in diarrheic or respiratory infections. In addition, the selected pathogens represent viruses and bacteria that may present diagnostic interference, thereby allowing evaluation of the assay’s specificity breadth. In this study, the recombinant plasmids pUC57-BDV-UTR and pUC57-EC-K99 were used as positive controls in all qPCR assays. Double-distilled water was used as a no-template negative control (NTC) to monitor potential contamination and nonspecific amplification. Positive and negative controls were included in each experimental run, including specificity testing, sensitivity evaluation, and clinical sample detection, to ensure the reliability and accuracy of the assay.

### Sensitivity testing of the multiplex qPCR assay

To determine the detection limit of the multiplex qPCR assay, recombinant plasmids pUC57-BDV-UTR and pUC57-EC-K99, containing the target gene fragments, were mixed at concentrations ranging from 10^6^–10^0^ copies/μL and subjected to 10-fold serial dilutions. The diluted samples were then evaluated using duplex quantitative PCR, and negative controls were included. All samples, including positive controls (recombinant plasmids) and negative controls (double-distilled water), were tested in triplicate.

### Validation of the multiplex qPCR assay using clinical samples

A total of 132 fecal samples were collected from neonatal calves at 17 independent cattle farms in Shandong Province, China. To minimize potential sampling bias, no more than 10 samples were collected from each farm. Among the collected samples, 69 were obtained from calves exhibiting clinical signs of diarrhea, while 63 were collected from non-diarrheic calves. All samples were subjected to routine laboratory diagnosis and were simultaneously used for clinical validation of the multiplex qPCR assay. Statistical analysis including kappa coefficient calculation was performed using SPSS version 26.0. The multiplex qPCR assay was applied to all clinical samples under identical experimental conditions. For specificity evaluation, laboratory-preserved positive samples of BVDV-1, BVDV-2, and *E. coli* K99, as well as positive clinical samples of nine other common bovine-related viruses and bacteria were included. In addition, plasmid standards (plasmids pUC57-BDV-UTR and pUC57-EC-K99 at 10^6^ copies/μL) were used as positive controls, while double-distilled water served as the negative control. Negative controls were included in each run to monitor background fluorescence and nonspecific amplification, and threshold settings were determined according to the instrument default parameters.

## Results

### Selection of primers and probes for the multiplex qPCR assay

**Fig 1:**
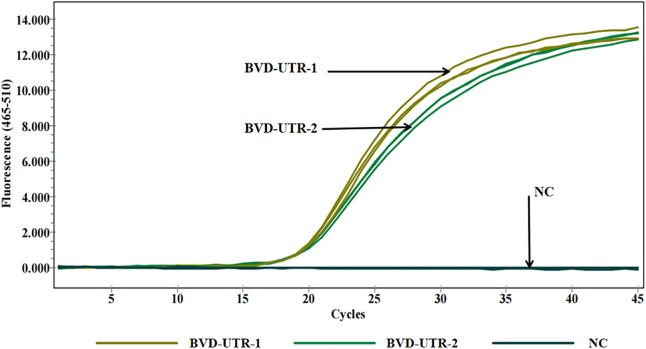
Amplification curves for BVDV primer pairs. Real-time qPCR amplification curves for BVDV primer pairs (BVD-UTR-1 and BVD-UTR-2) over 45 cycles. BVD-UTR-1 showed higher fluorescence intensity than BVD-UTR-2. NC: negative control.

**Fig 2:**
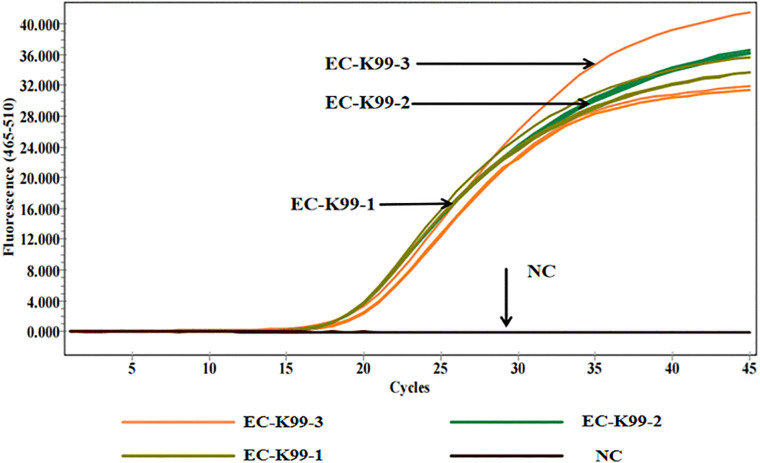
Amplification curves for *E. coli* K99 primer pairs. Real-time qPCR amplification curves for *E. coli* K99 primer pairs (EC-K99-1, EC-K99-2, and EC-K99-3) over 45 cycles. EC-K99-1 and EC-K99-2 showed higher fluorescence intensity than EC-K99-3. NC: negative control.

Two pairs of specific primers and probes were designed for the 5’ UTR region of BVDV, and three primer-probe pairs were designed for the K99 sequence of *E. coli*. Although the results in both Figs 1 and 2 indicate that no significant difference was observed between BVD-UTR-1 and BVD-UTR-2, BVD-UTR-1 demonstrated a higher amplification efficiency than BVD-UTR-2. Similarly, EC-K99-1 and EC-K99-2 exhibited a more optimal amplification efficiency than EC-K99-3. After a comprehensive comparison, both BVD-UTR-1 and EC-K99-1 were selected and used in all subsequent experiments for this study.

### Construction of a standard curve for the multiplex qPCR assay

**Fig 3:**
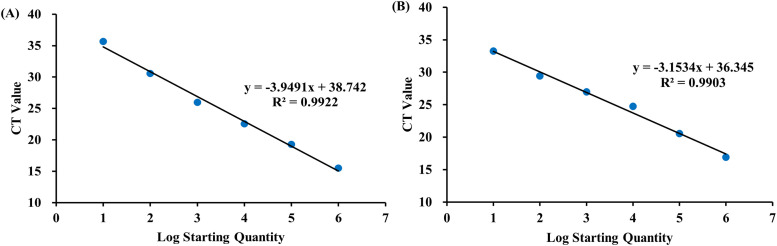
Standard curves for BVDV (A) and *E. coli* (B) generated using multiplex qPCR. Six-point standard curves were established using plasmids (10¹–10⁶ copies/μL) as templates, with negative controls included and triplicate testing for each sample. Ct values (Y-axis) were plotted against plasmid concentrations (X-axis) using Roche480 software. (A) The standard curve for BVDV shows a regression equation of y = −3.9491x + 38.742 with an R² value of 0.9922. (B) The standard curve for *E. coli* shows a regression equation of y = −3.1534x + 36.345 with an R² value of 0.9903.

We then performed a multiplex qPCR reaction using pUC57-BDV-UTR and pUC57-EC-K99 recombinant plasmid standards, with concentrations ranging from 10¹ to 10⁶ copies/μL. The obtained amplification curves and data were used to generate standard curves by plotting the log of the plasmid concentration (x-axis) against the Ct values (y-axis). This allowed for the evaluation of the specific amplification efficiency of the designed primer-probe sets. Regression analysis demonstrated a strong linear relationship between Ct values and log-transformed copy numbers for both targets, with R² values of 0.9922 for BVDV and 0.9903 for *E. coli* (Fig 3).

### Evaluation of the sensitivity of the multiplex qPCR assay

**Fig 4:**
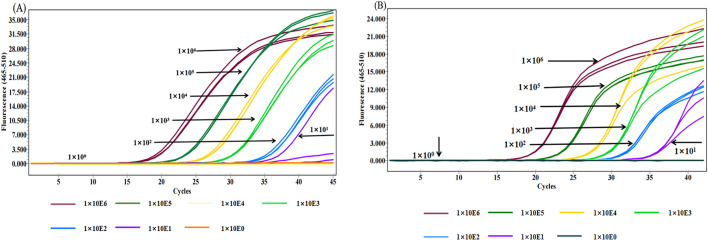
Analytical sensitivity of the multiplex qPCR for BVDV (A) and *E. coli* (B). A tenfold serial dilution of DNA ranging from 10⁶ to 10⁰ copies/μL was used to determine the lowest detectable concentration of target DNA. Amplification signals were observed across serial dilutions; however, the limit of detection was defined as 10² copies/μL for BVDV and 10¹ copies/μL for E. coli based on consistent amplification performance. Negative controls were included in each experiment, confirming assay specificity.

To evaluate the analytical sensitivity of the multiplex qPCR assay, we performed 10-fold serial dilutions of a recombinant plasmid mixture, ranging from 10^6^–10^0^ copies/μL. This dilution series was then utilized as a template for the multiplex qPCR assay to determine the lowest detectable concentration of the target DNA. The results indicated that the minimum detectable concentration was 10^2^ copies/μL for BVDV and 10^1^ copies/μL for *E. coli* DNA. Moreover, the amplification curves consistently demonstrated clear and distinct signals at this detection limit, confirming the robustness of the assay, even at very low DNA concentrations (Fig 4). This analytical sensitivity reflects the lower limit of detection of the assay under controlled experimental conditions. Because plasmid DNA standards were used in this study, the analytical sensitivity reflects the detection limit of the PCR amplification step rather than the reverse transcription efficiency for viral RNA.

### Evaluation of the specificity of the multiplex qPCR assay

**Fig 5:**
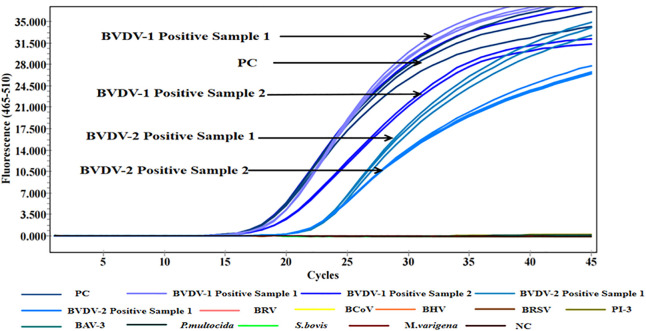
Specific amplification of BVDV by multiplex qPCR. Amplification curves for BVDV-1, BVDV-2, and positive controls (PC), alongside nine other bovine pathogens and negative control (NC), were generated to assess assay specificity. Only BVDV-positive samples showed amplification, confirming the high specificity of the multiplex qPCR without cross-reactivity.

**Fig 6:**
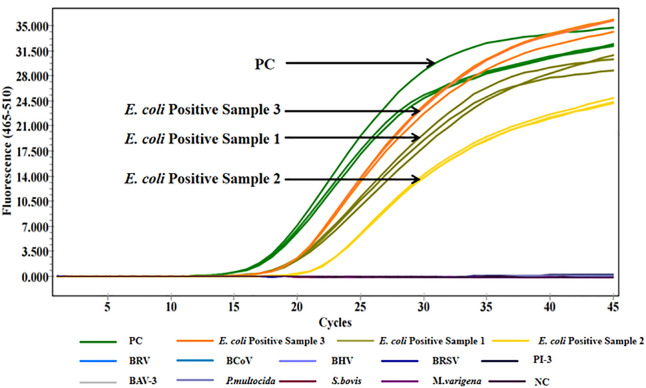
Specific amplification of *E. coli* by multiplex qPCR. Multiplex qPCR was performed on *E. coli* positive samples, positive control (PC), and nine non-target bovine pathogens. Distinct amplification was observed for *E. coli* and PC, while no amplification occurred for non-target pathogens or negative control (NC), confirming assay specificity.

Only the positive controls and positive clinical samples produced amplification signals in the multiplex qPCR assay. No amplification was detected in the negative controls or in reactions containing nucleic acids from nine non-target bovine-related viruses and bacteria, demonstrating high analytical specificity of the assay. Furthermore, the reliability of the assay was confirmed by its ability to differentiate between closely related pathogens, effectively minimizing false positives. These results demonstrate the suitability of the method for accurate pathogen detection in clinical diagnostics, supporting its application in real-world settings where specificity is crucial for reliable results (Figs 5 and 6). Furthermore, when applied to clinical samples, the multiplex qPCR results were fully consistent with those obtained using conventional diagnostic methods, supporting the diagnostic specificity of the assay. No amplification signals were detected in negative control reactions, supporting the reliability of the threshold setting used for result interpretation.

### Clinical validation of the multiplex qPCR assay

Using the established multiplex qPCR assay, 56 out of 132 clinical samples (42.4%) were positive for BVDV and/or *Escherichia coli* K99. Specifically, 34 samples were positive for BVDV, 27 samples were positive for *E. coli* K99, and 5 samples were positive for both pathogens, indicating coinfection. Among the diarrheic calves, 41 of 69 samples (59.4%) tested positive for at least one of the two pathogens, whereas 15 of 63 samples (23.8%) from non-diarrheic calves were positive. The detailed detection results for all 132 clinical samples, including the multiplex qPCR and conventional PCR/RT-PCR outcomes, are presented in S3 Table. The results obtained using the multiplex qPCR assay were fully consistent with those generated by conventional PCR methods (RT-PCR for BVDV and PCR for E. coli K99) (S4 Table), showing almost perfect diagnostic agreement in this sample set (kappa = 1.000, 95% CI: 1.000–1.000, P < 0.001), both reached 100% in this sample set. Accordingly, the positive predictive value (PPV), negative predictive value (NPV), and overall accuracy of the multiplex qPCR assay were all 100%.

## Discussion

NCD is one of the leading causes of mortality and morbidity in calves and accounts for more than 50% of the total calf deaths. NCD-induced calf deaths lead to significant economic losses and reduced productivity in the livestock industry [21]. The bacterial pathogens associated with calf diarrhea include *E. coli*, *Salmonella spp*., and *Clostridium perfringens*, and the highly contagious viral pathogen, BVDV. NCD can affect cattle of all breeds and ages, and as such, causes substantial economic losses worldwide [22]. Traditional pathogen identification methods, such as bacterial culture, virus isolation, and conventional PCR, are time-consuming and less suitable for rapid detection of mixed infections. In contrast, qPCR offers several advantages over conventional PCR, such as a lower cross-contamination risk, higher sensitivity, and the ability to conduct high-throughput analyses [23–25]. Multiplex PCR systems detect multiple target sequences simultaneously, usually by distinguishing amplicon sizes (traditional PCR) or using oligonucleotide probes labeled with different fluorophores (qPCR). These systems are reliable, cost-effective, and time-saving, and they are commonly applied in veterinary clinical diagnostics and surveillance programs [4,26]. In fact, multiple multiplex assays, both conventional and real-time PCR, have been developed to not only distinguish between pathogens causing clinically similar diseases in cattle [27,28], but also to monitor the spread of vector-borne viruses [29]. To our knowledge, few studies have reported a duplex real-time PCR assay simultaneously targeting an RNA viral pathogen and a bacterial virulence gene associated with neonatal calf diarrhea.

In this study, we developed a multiplex fluorescent qPCR method for the simultaneous detection of BVDV and *E. coli*. This method has significant potential in diagnostic applications. Since this assay can detect both BVDV and *E. coli* in a single reaction, it offers significant advantages over traditional methods in terms of time reduction, cost-efficiency, and increased throughput.

The analytical performance of the multiplex qPCR assay was evaluated using plasmid standards and primer–probe sets targeting conserved regions of BVDV and *Escherichia coli* K99. The assay demonstrated sufficient analytical sensitivity and specificity for reliable pathogen detection, without cross-reactivity with other bovine pathogens. In addition, strong concordance with conventional diagnostic methods further supported its diagnostic reliability. Compared with previously reported multiplex molecular assays for bovine pathogens, the present method achieved comparable analytical sensitivity while enabling simultaneous detection of viral and bacterial targets in a single reaction system. Specificity was further supported by both computational analysis and experimental validation. Primers and probes targeting the 5′ UTR region of BVDV and the conserved K99 gene region exhibited high specificity, which is particularly important for detecting coinfections under field conditions. The amplification efficiency of the duplex assay remained within an acceptable range for multiplex real-time PCR detection.

The reproducibility of the method was also evaluated. We found that both intra- and inter-assay variations were minimal, indicating that this multiplex qPCR assay provides consistent and reliable results across various conditions. This is critical for routine diagnostic testing, especially in clinical and research settings, where consistent and accurate results are essential for disease assessment and intervention.

We validated our qPCR assay using clinical samples. We found that the assay demonstrated consistent results with traditional diagnostic methods when tested with clinical samples of BVDV and *E. coli* from livestock farms, confirming the accuracy and reliability of the method in real-world applications. The ability to detect both BVDV and *E. coli* in a single reaction further enhances the practicality and efficiency of this method. A key advantage of the multiplex qPCR assay is its potential for high-throughput screening. In large-scale epidemiological studies, rapid detection of multiple pathogens is essential, and this multiplex qPCR method provides an efficient solution.

Thus, the multiplex fluorescent qPCR assay developed in this study provides a reliable, sensitive, and efficient diagnostic tool for the simultaneous detection of BVDV and *E. coli*. Its high specificity, sensitivity, and reproducibility make it suitable for both clinical and research applications, particularly in monitoring livestock health and preventing disease outbreaks. Future studies could expand the diagnostic capabilities of this assay by incorporating additional pathogen targets, providing a more comprehensive tool for veterinary diagnostics.

## Conclusions

This study successfully established a multiplex qPCR diagnostic assay capable of simultaneously detecting BVDV and *E. coli*. The developed method demonstrated suitable specificity and reproducibility for both BVDV and *E. coli*, with high efficiency and sensitivity, capable of detecting as low as 10^2^ copies/μL of BVDV RNA and 10^1^ copies/μL of *E. coli* DNA. Furthermore, this assay was further evaluated for precision, considering both intra- and inter-batch variability. Clinical samples of BVDV genotypes 1 and 2, as well as clinical *E. coli* samples, were tested and compared with positive controls, revealing that the multiplex qPCR method exhibited reliable analytical performance. In conclusion, the diagnostic approach developed in this study is highly reliable for the simultaneous detection of BVDV and *E. coli* in livestock. Thus, this assay provides a robust tool for farmers and regulatory authorities to monitor these pathogens, thereby helping to prevent significant economic losses and outbreaks of disease.

## Supporting information

S1 Table GenBank sequences used for primer and probe design of BVDV and *Escherichia coli* K99. (DOCX)

S2 Table Primer sequences used for conventional PCR and RT-PCR assays. (DOCX)

S3 Table Detailed results of multiplex qPCR and conventional PCR/RT-PCR detection in 132 clinical samples. (DOCX)

S4 Table Statistical comparison between multiplex qPCR and conventional PCR/RT-PCR for detection of BVDV and *Escherichia coli* K99. (DOCX)

S1 Fig Multiple sequence alignment of primer and probe binding regions for BVDV and *Escherichia coli* K99. Multiple sequence alignment of representative sequences used for primer and probe design. (A–C) Alignment of the binding regions of the forward primer, reverse primer, and probe for BVDV. (D–F) Alignment of the binding regions of the forward primer, reverse primer, and probe for Escherichia coli K99. The primer and probe binding regions are indicated by boxes. The alignment demonstrates that the selected primer–probe sets are located in highly conserved regions among different strains.
(TIF)
