# Supplementary material for: Development of a multiplex fluorescent qPCR assay for the simultaneous detection of bovine viral diarrhea virus and pathogenic Escherichia coli
Source: PLoS One. 2026 May 15;21(5):e0349315. doi: 10.1371/journal.pone.0349315 (PMC13178876; doi:10.1371/journal.pone.0349315)
Supplement: S1 Table — (DOCX) [file pone.0349315.s001.docx]

**S1 Table**. GenBank sequences used for primer and probe design of BVDV and Escherichia coli K99.

| **No.** | **Pathogen** | **Strain** | **Genotype/Gene** | **GenBank accession number** |
| --- | --- | --- | --- | --- |
| 1 | BVDV | CHN/HB-01/2017 | BVDV-1 | ON901784.1 |
| 2 | BVDV | NADL | BVDV-1 | NC_001461.1 |
| 3 | BVDV | Oregon C24V | BVDV-1 | AF091605.1 |
| 4 | BVDV | Osloss | BVDV-1 | M96687.1 |
| 5 | BVDV | TGAC 05 | BVDV-1 | PV626349.1 |
| 6 | BVDV | CC13B | BVDV-1 | KF772785.1 |
| 7 | BVDV | PZ33 | BVDV-1 | PP856402.1 |
| 8 | BVDV | 26/Australia/2021 | BVDV-1 | PV578941.1 |
| 9 | BVDV | BJ1201 | BVDV-1 | KT943518.1 |
| 10 | BVDV | 95-1501 | BVDV-2 | MH231130.1 |
| 11 | BVDV | JV14 | BVDV-2 | MH231136.1 |
| 12 | BVDV | 277 | BVDV-2 | PX122060.1 |
| 13 | BVDV | 108 | BVDV-2 | KF835701.1 |
| 14 | BVDV | 890 | BVDV-2 | NC_039237.1 |
| 15 | BVDV | NY'93/C | BVDV-2 | AF502399.1 |
| 16 | *E. coli* | reference strain | K99 | M35282.1 |
| 17 | *E. coli* | NX-K99 | K99 | FJ864678.1 |
| 18 | *E. coli* | 2NT | K99 | JX987524.1 |
| 19 | *E. coli* | O55 | K99 | MH916617.1 |
| 20 | *E. coli* | SHI-RAZI 88 | K99 | GU951525.1 |
| 21 | *E. coli* | BE311 | K99 | CP097214.1 |
| 22 | *E. coli* | CFS3246 | K99 | CP026930.2 |
| 23 | *E. coli* | E94 | K99 | CP119745.1 |
| 24 | *E. coli* | E92 | K99 | CP119739.1 |
| 25 | *E. coli* | O55 F5 | K99 | MF467447.1 |
| 26 | *E. coli* | J11 | K99 | KR870316.1 |
| 27 | *E. coli* | Mashhad-Iran K99 | K99 | KP054295.1 |
| 28 | *E. coli* | STLIN_6 | K99 | CP058791.1 |
| 29 | *E. coli* | J4 | K99 | KR606337.1 |
| 30 | *E. coli* | F5 | K99 | MF467448.1 |
