# Supplementary material for: Development of a multiplex fluorescent qPCR assay for the simultaneous detection of bovine viral diarrhea virus and pathogenic Escherichia coli
Source: PLoS One. 2026 May 15;21(5):e0349315. doi: 10.1371/journal.pone.0349315 (PMC13178876; doi:10.1371/journal.pone.0349315)
Supplement: S2 Table — (DOCX) [file pone.0349315.s002.docx]

**S2 Table**. Primer sequences used for conventional PCR and RT-PCR assays.

| **Target** | **Primer name** | **Sequence (5'-3')** | **Amplicon size** |
| --- | --- | --- | --- |
| BVDV | BVDV-F | TGACACCATCACCGACCAC | 84 bp |
|  | BVDV-R | CTCCCTCTGCCCATTTTT |  |
| *E. coli* K99 | K99-F | TATTATCTTAGGTGGTATGG | 314 bp |
|  | K99-R | GGTATCCTTTAGCAGCAGTATTTC |  |
