# Supplementary material for: Development of a multiplex fluorescent qPCR assay for the simultaneous detection of bovine viral diarrhea virus and pathogenic Escherichia coli
Source: PLoS One. 2026 May 15;21(5):e0349315. doi: 10.1371/journal.pone.0349315 (PMC13178876; doi:10.1371/journal.pone.0349315)
Supplement: S3 Table — (DOCX) [file pone.0349315.s003.docx]

**S3 Table**. Detailed results of multiplex qPCR and conventional PCR/RT-PCR detection in 132 clinical samples.

| **Sample_ID** | **Farm** | **Sample_type** | **Diarrhea** | **BVDV** | | ***E. coli* K99** | |
| --- | --- | --- | --- | --- | --- | --- | --- |
|  |  |  |  | **Multiplex qPCR** | **RT-PCR** | **Multiplex qPCR** | **PCR** |
| C01 | Farm1 | Feces | Yes | Positive | Positive | Negative | Negative |
| C02 | Farm1 | Feces | No | Positive | Positive | Negative | Negative |
| C03 | Farm1 | Feces | Yes | Positive | Positive | Negative | Negative |
| C04 | Farm1 | Feces | Yes | Negative | Negative | Positive | Positive |
| C05 | Farm1 | Feces | No | Negative | Negative | Negative | Negative |
| C06 | Farm1 | Feces | Yes | Negative | Negative | Negative | Negative |
| C07 | Farm1 | Feces | No | Negative | Negative | Negative | Negative |
| C08 | Farm1 | Feces | Yes | Negative | Negative | Negative | Negative |
| C09 | Farm2 | Feces | Yes | Positive | Positive | Positive | Positive |
| C10 | Farm2 | Feces | No | Positive | Positive | Negative | Negative |
| C11 | Farm2 | Feces | Yes | Positive | Positive | Negative | Negative |
| C12 | Farm2 | Feces | Yes | Positive | Positive | Negative | Negative |
| C13 | Farm2 | Feces | Yes | Negative | Negative | Positive | Positive |
| C14 | Farm2 | Feces | Yes | Negative | Negative | Positive | Positive |
| C15 | Farm2 | Feces | No | Negative | Negative | Negative | Negative |
| C16 | Farm2 | Feces | Yes | Negative | Negative | Negative | Negative |
| C17 | Farm2 | Feces | No | Negative | Negative | Negative | Negative |
| C18 | Farm2 | Feces | Yes | Negative | Negative | Negative | Negative |
| C19 | Farm3 | Feces | No | Negative | Negative | Negative | Negative |
| C20 | Farm3 | Feces | No | Negative | Negative | Negative | Negative |
| C21 | Farm3 | Feces | No | Negative | Negative | Negative | Negative |
| C22 | Farm3 | Feces | No | Negative | Negative | Negative | Negative |
| C23 | Farm3 | Feces | No | Negative | Negative | Negative | Negative |
| C24 | Farm3 | Feces | No | Negative | Negative | Negative | Negative |
| C25 | Farm3 | Feces | No | Negative | Negative | Negative | Negative |
| C26 | Farm3 | Feces | No | Negative | Negative | Negative | Negative |
| C27 | Farm3 | Feces | No | Negative | Negative | Negative | Negative |
| C28 | Farm4 | Feces | Yes | Positive | Positive | Positive | Positive |
| C29 | Farm4 | Feces | No | Positive | Positive | Negative | Negative |
| C30 | Farm4 | Feces | Yes | Positive | Positive | Negative | Negative |
| C31 | Farm4 | Feces | Yes | Positive | Positive | Negative | Negative |
| C32 | Farm4 | Feces | Yes | Negative | Negative | Positive | Positive |
| C33 | Farm4 | Feces | No | Negative | Negative | Negative | Negative |
| C34 | Farm4 | Feces | Yes | Negative | Negative | Negative | Negative |
| C35 | Farm4 | Feces | No | Negative | Negative | Negative | Negative |
| C36 | Farm4 | Feces | Yes | Negative | Negative | Negative | Negative |
| C37 | Farm5 | Feces | No | Positive | Positive | Negative | Negative |
| C38 | Farm5 | Feces | Yes | Positive | Positive | Negative | Negative |
| C39 | Farm5 | Feces | Yes | Positive | Positive | Negative | Negative |
| C40 | Farm5 | Feces | No | Negative | Negative | Negative | Negative |
| C41 | Farm5 | Feces | Yes | Negative | Negative | Negative | Negative |
| C42 | Farm5 | Feces | No | Negative | Negative | Negative | Negative |
| C43 | Farm5 | Feces | No | Negative | Negative | Negative | Negative |
| C44 | Farm6 | Feces | Yes | Positive | Positive | Negative | Negative |
| C45 | Farm6 | Feces | Yes | Positive | Positive | Negative | Negative |
| C46 | Farm6 | Feces | Yes | Negative | Negative | Positive | Positive |
| C47 | Farm6 | Feces | No | Positive | Positive | Negative | Negative |
| C48 | Farm6 | Feces | No | Negative | Negative | Negative | Negative |
| C49 | Farm6 | Feces | Yes | Negative | Negative | Negative | Negative |
| C50 | Farm6 | Feces | Yes | Negative | Negative | Negative | Negative |
| C51 | Farm6 | Feces | No | Negative | Negative | Negative | Negative |
| C52 | Farm7 | Feces | Yes | Positive | Positive | Negative | Negative |
| C53 | Farm7 | Feces | Yes | Positive | Positive | Negative | Negative |
| C54 | Farm7 | Feces | No | Positive | Positive | Negative | Negative |
| C55 | Farm7 | Feces | Yes | Negative | Negative | Positive | Positive |
| C56 | Farm7 | Feces | Yes | Negative | Negative | Negative | Negative |
| Note. Table continues on next page. | | | | | | | |
| C57 | Farm7 | Feces | No | Negative | Negative | Negative | Negative |
| C58 | Farm7 | Feces | Yes | Negative | Negative | Negative | Negative |
| C59 | Farm8 | Feces | No | Negative | Negative | Negative | Negative |
| C60 | Farm8 | Feces | No | Negative | Negative | Negative | Negative |
| C61 | Farm8 | Feces | No | Negative | Negative | Negative | Negative |
| C62 | Farm8 | Feces | No | Negative | Negative | Negative | Negative |
| C63 | Farm8 | Feces | No | Negative | Negative | Negative | Negative |
| C64 | Farm8 | Feces | No | Negative | Negative | Negative | Negative |
| C65 | Farm8 | Feces | No | Negative | Negative | Negative | Negative |
| C66 | Farm9 | Feces | Yes | Positive | Positive | Negative | Negative |
| C67 | Farm9 | Feces | No | Negative | Negative | Positive | Positive |
| C68 | Farm9 | Feces | Yes | Negative | Negative | Positive | Positive |
| C69 | Farm9 | Feces | No | Negative | Negative | Negative | Negative |
| C70 | Farm9 | Feces | Yes | Negative | Negative | Negative | Negative |
| C71 | Farm9 | Feces | No | Negative | Negative | Negative | Negative |
| C72 | Farm9 | Feces | No | Negative | Negative | Negative | Negative |
| C73 | Farm9 | Feces | Yes | Negative | Negative | Negative | Negative |
| C74 | Farm10 | Feces | Yes | Positive | Positive | Negative | Negative |
| C75 | Farm10 | Feces | No | Negative | Negative | Positive | Positive |
| C76 | Farm10 | Feces | Yes | Negative | Negative | Positive | Positive |
| C77 | Farm10 | Feces | Yes | Negative | Negative | Negative | Negative |
| C78 | Farm10 | Feces | No | Negative | Negative | Negative | Negative |
| C79 | Farm10 | Feces | No | Negative | Negative | Negative | Negative |
| C80 | Farm10 | Feces | Yes | Negative | Negative | Negative | Negative |
| C81 | Farm11 | Feces | Yes | Positive | Positive | Negative | Negative |
| C82 | Farm11 | Feces | No | Negative | Negative | Positive | Positive |
| C83 | Farm11 | Feces | Yes | Negative | Negative | Positive | Positive |
| C84 | Farm11 | Feces | Yes | Negative | Negative | Negative | Negative |
| C85 | Farm11 | Feces | No | Negative | Negative | Negative | Negative |
| C86 | Farm11 | Feces | Yes | Negative | Negative | Negative | Negative |
| C87 | Farm11 | Feces | No | Negative | Negative | Negative | Negative |
| C88 | Farm12 | Feces | Yes | Positive | Positive | Positive | Positive |
| C89 | Farm12 | Feces | Yes | Positive | Positive | Negative | Negative |
| C90 | Farm12 | Feces | Yes | Negative | Negative | Positive | Positive |
| C91 | Farm12 | Feces | No | Negative | Negative | Positive | Positive |
| C92 | Farm12 | Feces | No | Negative | Negative | Negative | Negative |
| C93 | Farm12 | Feces | Yes | Negative | Negative | Negative | Negative |
| C94 | Farm12 | Feces | Yes | Negative | Negative | Negative | Negative |
| C95 | Farm13 | Feces | Yes | Positive | Positive | Negative | Negative |
| C96 | Farm13 | Feces | Yes | Negative | Negative | Positive | Positive |
| C97 | Farm13 | Feces | No | Negative | Negative | Positive | Positive |
| C98 | Farm13 | Feces | No | Negative | Negative | Negative | Negative |
| C99 | Farm13 | Feces | Yes | Negative | Negative | Negative | Negative |
| C100 | Farm13 | Feces | No | Negative | Negative | Negative | Negative |
| C101 | Farm13 | Feces | Yes | Negative | Negative | Negative | Negative |
| C102 | Farm14 | Feces | Yes | Positive | Positive | Negative | Negative |
| C103 | Farm14 | Feces | Yes | Negative | Negative | Positive | Positive |
| C104 | Farm14 | Feces | No | Negative | Negative | Positive | Positive |
| C105 | Farm14 | Feces | No | Negative | Negative | Negative | Negative |
| C106 | Farm14 | Feces | No | Negative | Negative | Negative | Negative |
| C107 | Farm14 | Feces | Yes | Negative | Negative | Negative | Negative |
| C108 | Farm14 | Feces | Yes | Negative | Negative | Negative | Negative |
| C109 | Farm14 | Feces | No | Negative | Negative | Negative | Negative |
| C110 | Farm15 | Feces | Yes | Positive | Positive | Positive | Positive |
| C111 | Farm15 | Feces | Yes | Positive | Positive | Negative | Negative |
| C112 | Farm15 | Feces | Yes | Positive | Positive | Negative | Negative |
| C113 | Farm15 | Feces | Yes | Negative | Negative | Positive | Positive |
| C114 | Farm15 | Feces | Yes | Negative | Negative | Negative | Negative |
| C115 | Farm15 | Feces | Yes | Negative | Negative | Negative | Negative |
| Note. Table continues on next page. | | | | | | | |
| C116 | Farm15 | Feces | Yes | Negative | Negative | Negative | Negative |
| C117 | Farm16 | Feces | No | Positive | Positive | Negative | Negative |
| C118 | Farm16 | Feces | No | Negative | Negative | Positive | Positive |
| C119 | Farm16 | Feces | No | Negative | Negative | Negative | Negative |
| C120 | Farm16 | Feces | No | Negative | Negative | Negative | Negative |
| C121 | Farm16 | Feces | No | Negative | Negative | Negative | Negative |
| C122 | Farm16 | Feces | Yes | Positive | Positive | Negative | Negative |
| C123 | Farm16 | Feces | No | Negative | Negative | Negative | Negative |
| C124 | Farm17 | Feces | No | Negative | Negative | Positive | Positive |
| C125 | Farm17 | Feces | Yes | Positive | Positive | Positive | Positive |
| C126 | Farm17 | Feces | No | Negative | Negative | Negative | Negative |
| C127 | Farm17 | Feces | Yes | Positive | Positive | Negative | Negative |
| C128 | Farm17 | Feces | Yes | Negative | Negative | Positive | Positive |
| C129 | Farm17 | Feces | Yes | Negative | Negative | Negative | Negative |
| C130 | Farm17 | Feces | No | Negative | Negative | Negative | Negative |
| C131 | Farm17 | Feces | Yes | Negative | Negative | Negative | Negative |
| C132 | Farm17 | Feces | No | Negative | Negative | Negative | Negative |
