# Supplementary material for: Development of a multiplex fluorescent qPCR assay for the simultaneous detection of bovine viral diarrhea virus and pathogenic Escherichia coli
Source: PLoS One. 2026 May 15;21(5):e0349315. doi: 10.1371/journal.pone.0349315 (PMC13178876; doi:10.1371/journal.pone.0349315)
Supplement: S4 Table — (DOCX) [file pone.0349315.s004.docx]

**S4 Table**. Statistical comparison between multiplex qPCR and conventional PCR/RT-PCR for detection of BVDV and Escherichia coli K99.

| **Pathogen** | **Total samples** | **Multiplex qPCR positive (%)** | **Conventional PCR positive (%)** | **PPV (%)** | **NPV (%)** | **Accuracy (%)** | **Results of statistical analysis** | | |
| --- | --- | --- | --- | --- | --- | --- | --- | --- | --- |
|  |  |  |  |  |  |  | **Kappa Value** | **95% CI** | **P-value** |
| BVDV | 132 | 34 (25.8) | 34 (25.8) | 100 | 100 | 100 | 1.000 | 1.000–1.000 | <0.001 |
| *E. coli* K99 | 132 | 27 (20.5) | 27 (20.5) | 100 | 100 | 100 | 1.000 | 1.000–1.000 | <0.001 |

Note. PPV, positive predictive value; NPV, negative predictive value. All values were calculated based on comparison with conventional PCR/RT-PCR as the reference method.
